# Scaling of cardiovascular risk factors in 230 Latin American cities

**DOI:** 10.1038/s41598-025-92087-5

**Published:** 2025-03-01

**Authors:** Aureliano S. S. Paiva, Usama Bilal, Roberto F. S. Andrade, Claudiano C. Cruz Neto, J. Firmino de Sousa Filho, Gervásio F. Santos, Maurício L. Barreto, Daniel A. Rodriguez, Pricila Mullachery, Brisa Sanchez, Ana V. Diez-Roux, Felipe Montes, Andrés Trotta, Tania Alfaro, J. Jaime Miranda, Tonatiuh Barrientos-Gutierrez

**Affiliations:** 1 Center of Data and Knowledge Integration for Health (CIDACS), Fundação Oswaldo Cruz, Instituto Gonçalo Moniz, Salvador, Bahia Brazil; 2 Urban Health Collaborative, Department of Epidemiology and Biostatistics, Drexel Dornsife School of Public Health, Philadelphia, PA USA; 3 Institute of Physics, Federal University of Bahia, Salvador, Bahia Brazil; 4 Population Center for Agricultural, Environmental and Biological Sciences, Federal University of Recôncavo da Bahia, Cruz das Almas, Bahia Brazil; 5 Economics Faculty, Federal University of Bahia, Salvador, Bahia Brazil; 6 Department of City and Regional Planning and Institute of Transportation Studies, University of California Berkeley, Berkeley, CA USA; 7 Department of Industrial Engineering, Social and Health Complexity Center, Universidad de los Andes, Bogotá, Colombia; 8 Institute of Collective Health, Universidad Nacional de Lanús, Buenos Aires, Argentina; 9 Escuela de Salud Pública, Facultad de Medicina, Universidad de Chile, Santiago, Chile; 10 CRONICAS Center of Excellence in Chronic Diseases, Universidad Peruana Cayetano Heredia, Lima, Peru; 11 Department of Medicine, School of Medicine, Universidad Peruana Cayetano Heredia, Lima, Peru; 12 Center for Population Health Research, National Institute of Public Health, Cuernavaca, Mexico

**Keywords:** Urban health, Cardiovascular disease, Urban scaling, Proportion, Latin America, Applied physics, Statistical physics, thermodynamics and nonlinear dynamics, Risk factors

## Abstract

Urbanization results in increased numbers of people living in cities and poses challenges and opportunities to public health policies. Studies of urban scaling have unveiled how cities’ socio-economic and infrastructural attributes vary systematically with city size. Previous studies have explored the scaling properties of health outcomes across metropolitan areas in different countries, but chronic diseases have been infrequently examined. This paper examines scaling behaviors of 4 cardiovascular risk factors: hypertension, diabetes, tobacco smoking, and obesity across 230 cities in six countries of Latin America. In analyses pooled across countries, diabetes and hypertension showed weakly superlinear scaling (higher prevalence in larger cities). In comparison, obesity showed linear scaling, and tobacco showed weakly sublinear scaling (lower prevalence in larger cities), although most coefficients did not differ significantly from the null. In country-specific analyses, hypertension and diabetes tended to show a superlinear pattern across most countries, obesity tended to show a sublinear pattern in most countries, and tobacco tended to be superlinear (in contrast to the analysis pooled across countries where it was sublinear). Results suggest the need to examine further the drivers of this varying scaling of risk factors.

**Supplementary Information:**

The online version contains supplementary material available at 10.1038/s41598-025-92087-5.

## Introduction

More than half of the world’s population currently lives in cities. By 2050, this proportion is expected to reach 70%^1^. This concentration of people living in dense areas poses several challenges and opportunities on a global scale. For this reason, urban conditions are important components of the 2030 Sustainable Development Goals, as reflected in Goal 11: to make cities and human settlements inclusive, safe, resilient, and sustainable. In addition to its impact on social and economic interactions^2^, urban areas are often characterized by high incidence of chronic health conditions like cancer, cardiovascular, and chronic respiratory diseases, which coexist with infectious diseases such as HIV/AIDS and arboviruses^3,4^. There is an urgent need to understand the health consequences of this rapid urbanization process and how it can be managed to improve population health. Indeed, the linkages between urbanization and health are especially relevant in Latin America, where more than 80% of the population lives in cities, with Argentina having the highest urbanization rate at 92% and Peru the lowest at 78%, and where levels of social inequality are amongst the highest in the world^1^.

Urbanization can have specific impacts on numerous health outcomes, both beneficial and adverse. Urban living can affect health through many mechanisms, including environmental exposure, lifestyle, and access to healthcare. Understanding cities as “living organisms of different sizes” subject to the effects of scaling can lead to insights regarding how the functioning of urban systems shapes population health^5^. Prior studies on scaling have shown that features of cities, such as electric grids, streets, and social networks, display fractal structures similar to biological systems like capillaries or pulmonary systems^6^. However, less effort has been directed toward understanding how city population health changes with city size^7^. Investigating the scaling properties of city health outcomes can reveal how urbanization influences health risks and resource needs, suggesting ways to optimize the allocation of public health resources^8–10^. For example, examining how community energy expenditure and waste production scale with urban size and their links to health can inform necessary policies and interventions as cities grow. Similarly, understanding where disease rates are likely to be higher or lower can lead to better-targeted public health strategies^11^.

Urban scaling refers to how attributes of cities, such as infrastructure (water, sewage, electricity networks, transportation systems), services and economic productivity, social relations, and health outcomes change systematically as a function of city population size. Many of these attributes do not scale linearly, i.e., they can increase slower (sublinear scaling) or faster (superlinear scaling) than the population. Scaling (i.e., the relationship between size and other features) is a property of natural and human-developed systems; e.g., Galileo used scaling arguments to explain why large mammals have, comparatively, wider paws than smaller ones^12^. Scaling properties of living organisms, usually called allometric relations, have also been identified in other contexts, such as the relationship between metabolic rates and organism mass, summarized by the well-known sub-linear 3/4 Kleiber law dating from the 1930s^13^. Significant advances in understanding the origins of scaling laws in bio-systems came from the realization that fractal structures in living systems, such as the capillary or pulmonary systems, optimize energy delivery and thus reduce the required rate of energy expenditure per unit of mass^11,14^.

Over the last decade, limited studies have examined the scaling properties of population health outcomes. These studies were predominantly focused on AIDS cases, traffic-related injuries, and violent crime^3,4,7,8,15^, and on data from single countries or even regions. In a recent study^16^, we investigated the scaling properties of human mortality in US and Latin American cities. In contrast to results for US cities where all-cause mortality is lower in larger cities, we found no clear relation between city population and all-cause mortality in Latin American cities. We also found that cardiovascular diseases, the leading cause of death worldwide, scaled differentially between the US (sublinear scaling), Mexico (superlinear scaling), and the nine other Latin American countries in the study (linear scaling)^16^.

In this study, we build on prior work by examining how city size is related to the prevalence of cardiovascular disease risk factors. Latin America is a unique setting for the examination of urban scaling because more than 80% of its population lives in urban areas, a transition that has been taking place since the second half of the 20th century^17^. The region includes a mix of megacities and rapidly growing small to mid-sized cities, offering diverse environments to explore the links between city size and health. We make a novel contribution by seeking to go beyond the obtained mortality scaling behavior of non-communicable diseases (NCD) and detail the corresponding counterpart of their major risk factors—diabetes, hypertension, obesity, and tobacco. We base our investigation on a sub-set of the previously used harmonized data^16^, which details the specific risk factors of 230 cities across six Latin American countries: Argentina, Brazil, Chile, Colombia, Mexico, and Peru (see the Supplementary Materials). In addition, we provide access to information that reveals detailed insights by sex.

We focused on cardiovascular risk factors for several reasons: (a) risk factors may be more sensitive to environmental conditions than mortality which is the downstream consequence of risk factors but is also affected by other processes such as access to quality care; (b) cardiovascular diseases are the leading cause of death in most cities of the region^18^; (c) some Latin America countries have implemented robust policies to tackle some of these risk factors, including obesity and its related conditions (e.g., diabetes)^19–21^. This work aims to identify whether these risk factors exhibit sublinear, linear, or superlinear scaling with city size, thus providing more detailed insights into urban health dynamics. We hypothesize that the four risk factors will exhibit superlinear scaling as they will be more prevalent in larger cities. The hypothesis assumes that larger cities create more opportunities for interactions among people, which can increase the spread of behaviors or conditions, such as smoking, obesity, or hypertension.

We advance on previous work in three ways. First, by examining the scaling behavior of cardiovascular risk factors across multiple Latin American cities, we provide a broad and comparative perspective on how urbanization impacts health across the region, making our findings more generalizable. Second, while previous studies have focused primarily on mortality or limited health outcomes, our work focuses on key NCD risk factors, offering a more nuanced understanding of urban health dynamics. Third, we examine these risk factors in relation to population size while considering differences in country-specific healthcare systems and public health interventions, which adds depth to the analysis of urban health in Latin America. These innovations provide insights into how public health policies should address the evolving challenges posed by rapid urbanization in diverse and often unequal urban landscapes. Understanding the scaling properties of these risk factors helps identify strategies to mitigate health risks in cities of varying sizes and supports generating a hypothesis as an initial step in exploring scaling laws of CVD health outcomes. By establishing the fundamental relationship between city size and cardiovascular risk factors, we provide a baseline for future research. Subsequent studies with a causal goal in mind can incorporate environmental and cultural variables to build on our foundational work and offer more granular insights into the specific determinants of health in urban settings.

## Results

We examined the scaling behavior of the four risk factors for the female, male, and both combined (the entire population). Plots of results are displayed in supplementary material (Fig. SM2). The plots also show the best linear fit with the exponent (slope), its confidence interval, and R-squared values. We found a good fit to the data for the four risk factors, and the R-squared values are greater than 0.81 (except for tobacco for females and the entire population), indicating that the total population explains a significant amount of the variation on the count of persons with each risks factor. The R-squared values for tobacco, 0.767 for the entire population and 0.698 for the female population, are lower than those for other risk factors in this study. In the context of scaling theory, these lower R-squared values suggest that the scaling model explains less variability in tobacco prevalence than other risk factors.

In Table 1, the scaling coefficient analysis reveals significant associations for specific risk factors by sex. Notably, the coefficient for diabetes among females is 1.049 (95% CI: 1.001, 1.097). It suggests that the prevalence of diabetes increases slightly faster than the population size among females. Similarly, the coefficient for hypertension among females is 1.045 (95% CI: 1.011, 1.080), also showing a statistically significant positive association. For males, the coefficient for tobacco is 0.962 (95% CI: 0.926, 0.999), indicating a negative association. This result suggests that the prevalence of tobacco decreases as the population size increases among males.

Other coefficients, while not statistically significant, provide additional context. The coefficients for diabetes (1.008, 95% CI: 0.941, 1.076) and hypertension (1.022, 95% CI: 0.960, 1.085) in the overall population suggest a near-linear (or slightly superlinear) relationship with population size. Obesity shows a slight sublinear trend overall (0.993, 95% CI: 0.942, 1.045) and for both males (0.999, 95% CI: 0.942, 1.056) and females (0.990, 95% CI: 0.940, 1.040), indicating that its prevalence increases roughly in proportion to population size, as do the coefficients for diabetes and hypertension for males. Tobacco, with an overall coefficient of 0.930 (95% CI: 0.858, 1.003), indicates a slower increase compared to population growth, aligning with the statistically significant trend observed in males.

In summary, these results highlight sex-specific differences in scaling certain health risk factors with population size for Latin American cities. Counts of diabetes and hypertension increase faster than the population size among females, while counts of tobacco users increase less than the population size among males.

**Table 1:** Scaling coefficients by risk factor, overall and by sex, unadjusted model.

	Overall	Males	Females
Diabetes	1.008 (0.941, 1.076)	1.025 (0.977, 1.073)	1.049 (1.001, 1.097)
Hypertension	1.022 (0.960, 1.085)	1.045 (0.997, 1.093)	1.045 (1.011, 1.080)
Obesity	0.993 (0.942, 1.045)	0.999 (0.942, 1.056)	0.990 (0.940, 1.040)
Tobacco	0.930 (0.858, 1.003)	0.962 (0.926, 0.999)	0.958 (0.876, 1.040)

Analyses stratified by sex and adjusted for country can provide more detailed insights into the scaling behavior of the four cardiovascular risk factors, as in Table 2 and Figure SM3 in the supplementary material.

The scaling coefficients for diabetes, hypertension, obesity, and tobacco reveal notable country-specific and sex-specific patterns. For diabetes, Chile exhibits a significant superlinear relationship for the entire population with a coefficient of 1.055 (95% CI: 1.00, 1.11). Mexico shows a similar significant trend among males, with a coefficient of 1.034 (95% CI: 1.006, 1.062). Additionally, Chilean males showed a significant superlinear relationship with a coefficient of 1.091 (95% CI: 1.003, 1.179).

The data from Mexico reveal a significant superlinear relationship among females regarding tobacco, with a coefficient of 1.123 (95% CI: 1.047, 1.199), indicating a faster increase in tobacco prevalence among women. The data suggests that diabetes and tobacco exhibit predominantly superlinear scaling for the populations, while obesity shows mostly a sublinear trend.

**Table 2:** Scaling coefficients overall and by sex (95% confidence intervals) for cardiovascular risk factors by country.

	Country*	Diabetes	Hypertension	Obesity	Tobacco
EP	AR (2013)	1.030 (0.974, 1.086)	1.010 (0.966, 1.053)	0.985 (0.937, 1.034)	1.015 (0.979, 1.052)
	BR (2013)	1.002 (0.956, 1.047)	1.023 (0.967, 1.079)	0.989 (0.947, 1.031)	1.030 (0.974, 1.087)
	CL (2017)	1.055 (1.00, 1.11)	1.028 (0.906, 1.150)	0.961 (0.914, 1.009)	1.024 (0.957, 1.090)
	CO (2007)	0.983 (0.919, 1.047)	1.011 (0.943, 1.079)	0.982 (0.914, 1.050)	1.041 (0.964, 1.118)
	MX (2016)	1.008 (0.985, 1.031)	0.996 (0.946, 1.047)	0.999 (0.976, 1.023)	1.039 (0.995, 1.083)
	PE (2016)	1.015 (0.917, 1.112)	0.999 (0.901, 1.097)	1.021 (0.893, 1.149)	1.038 (0.936, 1.140)
FP	AR (2013)	1.025 (0.961, 1.088)	1.009 (0.955 1.064)	0.988 (0.928, 1.047)	1.026 (0.951, 1.102)
	BR (2013)	0.990 (0.940, 1.039)	1.016 (0.960, 1.072)	1.010 (0.966, 1.053)	1.066 (0.967, 1.164)
	CL (2017)	1.030 (0.955, 1.106)	1.056 (0.962, 1.149)	0.966 (0.910, 1.022)	1.009 (0.909, 1.109)
	CO (2007)	1.005 (0.939, 1.071)	0.994 (0.932, 1.056)	1.003 (0.932, 1.073)	1.027 (0.910, 1.144)
	MX (2016)	0.988 (0.949, 1.027)	0.980 (0.931, 1.029)	0.995 (0.969, 1.022)	1.123 (1.047, 1.199)
	PE (2016)	1.035 (0.915, 1.155)	0.991 (0.877, 1.106)	0.987 (0.874, 1.101)	1.107 (0.978, 1.236)
MP	AR (2013)	1.034 (0.956, 1.112)	1.011 (0.967, 1.054)	0.985 (0.930, 1.040)	1.009 (0.979, 1.039)
	BR (2013)	1.016 (0.965, 1.068)	1.035 (0.965, 1.105)	0.964 (0.908, 1.020)	1.009 (0.961, 1.057)
	CL (2017)	1.091 (1.003, 1.179)	0.996 (0.820, 1.172)	0.957 (0.904, 1.010)	1.032 (0.965, 1.098)
	CO (2007)	0.954 (0.876, 1.033)	1.036 (0.941, 1.131)	0.951 (0.863, 1.040)	1.046 (0.973, 1.118)
	MX (2016)	1.034 (1.006, 1.062)	1.024 (0.957, 1.091)	1.005 (0.965, 1.045)	1.015 (0.968, 1.052)
	PE (2016)	0.989 (0.894, 1.084)	1.007 (0.906, 1.108)	1.071 (0.915, 1.227)	1.022 (0.916, 1.128)

*AR (Argentina), Brazil (BR), Chile (CL), Colombia (CO), Mexico (MX), Peru (PE).

While not statistically significant, the remaining coefficients provide insights into the relationship between risk factors and population size. For diabetes, Argentina and Brazil generally show near-linear relationships for both the entire population and by sex, with coefficients hovering around 1. Specifically, the coefficients for the entire population are 1.030 (95% CI: 0.974, 1.086) for Argentina and 1.002 (95% CI: 0.956, 1.047) for Brazil. Similar near-linear trends are observed for females and males in these countries.

Hypertension shows slight but not significant trends towards superlinear scaling in Chile (2017) for females (coefficient 1.056, 95% CI: 0.962, 1.149) and in Brazil (2013) for males (coefficient 1.035, 95% CI: 0.965, 1.105). Both Argentina and Brazil indicate near-linear relationships for the entire population. Obesity trends towards a sublinear relationship in Argentina for the entire population (coefficient 0.985, 95% CI: 0.937, 1.034) and for males in Brazil (coefficient 0.964, 95% CI: 0.908, 1.020). Near-linear relationships are observed for females in both countries. Tobacco presents slight superlinear trends in Brazil (2013) (coefficient 1.030, 95% CI: 0.974, 1.087) and Colombia (2007) (coefficient 1.041, 95% CI: 0.964, 1.118) for the entire population. Near-linear relationships are observed in Argentina (2013) and Brazil (2013) for the male population.

## Discussion

### Key findings

Our analyses of the scaling properties of cardiovascular risk factors in Latin American cities yielded three key findings. First, in our study of 230 cities, diabetes, and hypertension showed weakly superlinear scaling (higher prevalence in larger cities), while tobacco and obesity displayed weakly sublinear scaling (lower prevalence in larger cities). Among 12 sets of results (four risk factors, two genders, and the entire population), we found that coefficients tended to be superlinear for hypertension and diabetes but linear or sublinear for obesity and tobacco, with similar patterns for men and women. Tobacco showed the most consistent sublinear relationships. Three statistically significant coefficients stand out among this set of results: superlinear scaling coefficients for diabetes and hypertension in women and a sublinear scaling for tobacco in men.

Second, in country-specific analyses, hypertension and diabetes tended to show a superlinear pattern across most countries, obesity tended to show a sublinear pattern in most countries, and tobacco tended to be superlinear (in contrast to the analysis pooled across countries where it was sublinear). Notably, coefficients for tobacco were superlinear in men and women, with slightly larger coefficients in women. However, scaling coefficients significantly differed from one in only four of the 72 combinations of six countries, four risk factors, and three population sets.

Third, though country-specific scaling coefficients have some similarities, there were also some patterned differences across countries. The coefficients for Argentina and Brazil exhibit very similar patterns. Both countries have superlinear coefficients for diabetes, hypertension, and tobacco in the entire population and in the male population. In Mexico, the female population shows a distinct pattern, where almost all scaling coefficients are sublinear, except for tobacco, which is superlinear, and all coefficients were superlinear for men. Among the four cardiovascular risk factors analyzed, obesity predominantly exhibited a sublinear behavior across the countries and groups studied. This sublinear scaling of obesity contrasts with the other risk factors.

### Comparison with prior studies

Prior studies exploring the scaling of health outcomes have found heterogeneous results^9^. Patterson-Lomba et al.^15^ have seen a superlinear scaling phenomenon for sexually transmitted infections in US Metropolitan Areas, where relatively more cases of sexually transmitted diseases were observed in bigger cities, meaning that they occur at a higher rate in larger cities. In another study, Rocha et al.^22^ examined a wider variety of outcomes, including HIV, infant mortality, diabetes, heart attack, and colon cancer in US counties, as well as in Swedish and Brazilian municipalities^22^. They suggest that, while the scaling of infectious diseases may be related to network effects and the provision of sanitation services, the causal chain of chronic diseases may be more complex and involve factors that may scale in different ways with urban size – pollution, traffic, exposure to ultra-processed foods, social norms, primary care, and intensive care health assistance, etc. They found that obesity (USA and Sweden), smoking (USA), physical inactivity (USA), and limited access to healthy food (USA) all scale sub-linearly with population size, meaning that they increase at a slower rate than the population.

In a previous study, we found that cardiovascular disease scaled sublinearly in US cities and linearly in Latin American cities overall, except for Mexico, where it scaled superlinearly^16^. The linear scaling of cardiovascular disease deaths is generally consistent with our results, which fail to reject the null of linearity for risk factors. On the other hand, we speculate that the more robust superlinear scaling of smoking in females in Mexico may be related to the more superlinear scaling of cardiovascular diseases in Mexico, given the strength of the smoking-cardiovascular mortality association^23^. In another study involving multiple outcomes in the US, Brazilian, and Swedish cities, heart attack mortality scaled superlinearly in Brazil and sub-linearly in Sweden^22^. Thus, the general trend of our findings, indicating a relatively weak superlinear scaling behavior for CVD risk factors, is in line with these previous analyses.

These cross-country comparisons of CVD risk factors are especially relevant, revealing that, although Latin American countries face several similar problems, existing socio-economic and cultural singularities may give rise to detectable albeit minor differences in the scaling properties. Understanding the scaling behaviors of cardiovascular risk factors, as opposed to just cardiovascular mortality, may allow for the understanding of the linkages between the urbanization process and the first cause of death in Latin American cities. Scaling differences across countries may depend on the scaling of urban factors such as pollution, traffic, and ultra-processed foods as well as the scaling properties of healthcare-related factors, like the network of primary care and intensive care health assistance, which may favor larger cities more strongly in less developed countries^24,25^.

### Gender differences

We emphasize that deep-seated cultural and social issues influence gender disparities in accessing healthcare in Latin America^26^. Our findings revealed significant differences in the risk factors for cardiovascular prevalence between men and women. For men, cultural norms related to machismo often discourage them from seeking professional help for health issues, leading to underreported and unreliable health data^27^. This cultural barrier, combined with existing systemic barriers to healthcare access, means that men’s health issues are often neglected or insufficiently addressed. Consequently, men are less likely to engage in preventive care and more likely to delay seeking treatment until conditions become severe, exacerbating health outcomes and increasing healthcare costs^27–29^.

The challenges are multifaceted for women, rooted in cultural and structural inequalities^30^. Women in Latin America, especially those in vulnerable situations, face significant barriers to accessing health care due to lower job opportunities and income levels^31^. The high proportion of women working in the informal sector exposes them to unsafe working conditions, including the risk of sexual harassment^30^. It often leaves them without the protection of labor laws, social benefits, health insurance, or paid sick leave^29^. These conditions limit women’s ability to afford and access necessary health services, contributing to poorer health outcomes, particularly for chronic diseases such as cardiovascular disease (CVD).

The intersection of gender with economic and social vulnerabilities underscores the need for targeted public health policies in Latin America. For men, this includes promoting health-seeking behaviors and reducing the stigma associated with seeking medical help^27^. For women, it involves protecting informal workers, expanding access to health insurance, and implementing measures to combat gender-based violence and harassment^30^. Therefore, the observed differences by sex in cardiovascular risk factors may be partly driven by how these structural and cultural elements are magnified in larger cities. Future studies could benefit from explicitly examining how urbanization modulates these factors, offering a clearer understanding of the interplay between city size, gender, and health outcomes.

### Implications

In our unadjusted model, diabetes and hypertension show tendencies towards superlinear scaling, especially for females. This suggests that larger cities may have a higher prevalence of these conditions, possibly due to stress, lifestyle changes, or access to healthcare services that vary with city size^32,33^. Obesity does not show an apparent deviation from linear scaling, implying that its prevalence is relatively consistent regardless of city size. This might indicate uniform exposure to risk factors like diet and physical activity levels across cities of different sizes. Tobacco shows a sublinear scaling trend, particularly significant for males, indicating that tobacco prevalence tends to be lower in larger cities. This could be due to stricter tobacco regulations, higher health awareness, or better access to tobacco cessation programs in larger urban areas^34^.

In the country’s stratified model, diabetes and tobacco exhibit prominent superlinear scaling. Hypertension also shows superlinear tendencies but to a lesser extent. Interestingly, obesity consistently demonstrates sublinear behavior, indicating a slower increase in prevalence relative to population size. This pattern might reflect the more uniform exposure to obesity-related risk factors or better urban resources to counteract obesity. The similarities in the coefficients for Argentina and Brazil highlight comparable urban health dynamics. At the same time, Mexico shows a distinct pattern with predominantly sublinear coefficients for females, except for tobacco.

The consumption of ultra-processed foods, often associated with obesity, may be more evenly distributed across cities regardless of size. However, larger cities may offer more opportunities for healthier choices, particularly for those with greater socioeconomic means. This contrasts with the superlinear scaling of diabetes and hypertension, where city growth likely exacerbates stress, sedentary lifestyles, and healthcare disparities, leading to higher prevalence rates. Therefore, the sublinear trend in obesity may reflect a more complex interaction between urbanization, public health policies, and individual behaviors, warranting further investigation into the underlying mechanisms linking city growth to CVD risk factors.

The differences in scaling behaviors of cardiovascular risk factors across countries may be influenced by varying public health policies, healthcare infrastructure, and socioeconomic conditions^26^, as well as changing relationships between city size and other urban factors influencing risk factor prevalences. Brazil’s robust anti-smoking laws and the Family Health Strategy have significantly improved access to health services, particularly in urban areas, leading to better management of chronic diseases like diabetes and hypertension^35^. Similarly, with its high urbanization rate and extensive healthcare system, Argentina has made strides in mitigating urban health risks, although regional disparities persist^36^. Chile’s public health initiatives, such as food labeling and sugar taxes, have influenced distinct patterns in obesity management, reflecting the effectiveness of these recent policies^37^. In contrast, Peru’s less comprehensive healthcare coverage highlights the challenges in managing chronic diseases in its urban areas, where the healthcare system struggles to meet the growing demand^38^. With its intermediate urbanization and evolving healthcare system, Colombia has seen varied success in improving access and quality of care, with ongoing efforts to reduce the prevalence of risk factors^39^. Despite its extensive urbanization, Mexico faces challenges in achieving uniform healthcare outcomes, with public health initiatives showing mixed results across different regions and population groups^26^.

### Strengths and limitations

We could estimate scaling coefficients for a sample in cities of six different countries. While estimates of prevalences are model-based, we harmonized high-quality national surveys to make results comparable across countries. In scaling analysis, we compute both the scaling coefficient (β) and the pre-factor (α), as indicated in Eq. (1). However, the primary focus is on the scaling exponent β because it reveals how the prevalence of health outcomes changes with city size. This coefficient is crucial for understanding the proportional changes in health outcomes relative to the urban population. While the pre-factor α sets the baseline level of the outcome, β provides deeper insights into the dynamics of urbanization and its impact on health metrics, making it the key focus of our analysis and discussion. The baseline value α may reflect some regional influence of city properties that may or may not interfere with the scaling behavior for different sets of cities. For instance, sets of cities of two other countries with varying values of α_1_ and α_2_ (>α_1_) but the same β show the same scaling behavior. Still, the number of individuals affected by the outcome in country 2 is more prominent than its counterpart in country 1.

We want to call attention to potential caveats to our findings. First, these countries may be in different stages of the epidemiologic transition^40^, creating heterogeneities in the levels of CVD risk factors. Second, scaling behaviors may be sensitive to the definition of cities^41^. However, we were unable to test different definitions. Third, regional or country differences in the history of urban systems may make comparisons of cities of similar size across countries challenging. Our prior study examining the scaling of mortality, including CVD, found evidence of heterogeneity across the region^16^. Our cross-sectional analysis precludes any longitudinal interpretation in terms of city growth. This implies ergodicity or the assumption that cross-sectional and longitudinal population changes would render similar results, which is an unproved assumption that is problematic^42,43^. Finally, our estimates of risk factor prevalences are modeled and may sometimes be based on small and not fully representative samples. In addition, risk factors are self-reported, and for several (hypertension and diabetes), their diagnosis is likely influenced by access to health care, which may itself be patterned by city size. Of note, only a few results in Table 2 are statistically significant, and small numbers of cities render country-specific results highly variable and possibly unreliable.

It is important to note that the descriptive analysis of scaling properties is not intended to draw causal inferences and, therefore, is not designed to conclude the causal effect of city size on outcomes. Hence, adjustment for confounders is not needed. However, the description of scaling properties can raise important questions and provide insights into dynamics linking urban areas to health^44,45^. Instead, it simplifies the model to focus on the relationship between population size and health risk factors. Cultural factors are also important in shaping health behaviors and outcomes. Social, economic, environmental, and cultural factors are critical to city health but are not directly investigated in scaling studies like ours. Scaling law models are based on the principle that many urban characteristics, including health outcomes, exhibit predictable patterns as city populations grow^46,47^. These models have been extensively validated in various contexts, showing that the primary variable of interest - city size - captures a significant portion of the variance in urban attributes^6,45^.

## Methods

### Study setting

This study is part of the SALURBAL (*Salud Urbana en América Latina*, in the original Spanish acronym) Project, an international collaboration investigating the health impacts of urban policies and urban environment features in 371 cities across 11 countries in Latin America and the Caribbean. SALURBAL ‘cities’ include either a single administrative unit (e.g., a municipality) or a cluster of adjacent units (e.g., multiple municipalities) encompassing an urban agglomeration with a total population of at least 100,000 residents as of 2010^48,49^.

The population size is based on 2010 census estimates or, when unavailable, extrapolated numbers based on population counts from the previous census. Details on city definitions can be found elsewhere^48^. National risk factors surveys obtained information on the prevalence of risk factors (Table 3). Because some cities lack information on certain risk factors for specific years, we limited the analysis to 230 cities across six countries to ensure consistency in comparing scaling properties for different risk factors. More details are available in Table 3.

Risk factor data were obtained from national surveys conducted for risk factor surveillance. In Argentina, the 2013 *Encuesta Nacional de Factores de Riesgo* (ENFR-AR) focused on identifying risk factors for NCDs among the Argentine population using standardized questionnaires. Brazil’s 2013 *Pesquisa Nacional de Saúde* (PNS-BR) aimed to evaluate the health status of Brazilians, including the prevalence of key NCD risk factors, using interviews and physical examinations. Chile’s 2017 *Encuesta Nacional de Salud* (ENS-CL) collected detailed information on the population’s health conditions and behaviors. This survey used a combination of self-reported data and clinical measurements. Colombia’s 2010 *Encuesta Nacional de Salud* (ENS-CO) and Mexico’s 2016 *Encuesta Nacional de Salud y Nutrición* (ENSANUT-MX) focus on health status. These surveys combined detailed dietary assessments, health interviews, and physical measurements. Peru’s 2016 *Encuesta Demográfica y de Salud Familiar* (ENDES-PE) collected demographic and health data.

**Table 3:** Information on the analyzed data.

Country	Source* and year	Number of cities	Sample of people included in the study
Argentina	ENFR, 2013	33	21,451
Brazil	PNS, 2013	27	29,353
Chile	ENS, 2017	21	2,840
Colombia	ENSIN, 2010	35	18,824
Mexico	ENSANUT, 2016	91	26,335
Peru	ENDES, 2016	23	12,597

*Encuesta Nacional de Factores de Riesgo (ENFR-AR), Pesquisa Nacional de Saúde (PNS-BR); Encuesta Nacional de Salud (ENS-CL), Encuesta Nacional de Salud (ENS-CO), Encuesta Nacional de Salud y Nutrición, (ENSANUT-MX), Encuesta Demográfica y de Salud Familiar (ENDES-PE).

### Data sources

#### Survey variables definition and harmonization

The primary outcomes investigated are the number of people with cardiovascular risk factors by city of residence at the time of the cross-sectional surveys among adults 20–89. The counts were derived by multiplying estimated prevalences by the population. The prevalence of diabetes, hypertension, obesity, and tobacco were derived from national surveys in each country. Table 4 shows the summary statistics of the population proportion for each risk factor.

**Table 4:** City-level prevalence estimates for selected risk factors in 230 cities in six Latin American countries.

Risk factor	Mean	Standard error	Min	25%	50%	75%	Max
Diabetes	7.1	2.8	2.1	4.3	7.6	9.4	13.5
Hypertension	12.0	4.4	3.1	8.0	11.1	15.5	24.4
Tobacco	18.6	7.2	8.6	13.0	16.7	24.2	38.2
Obesity	27.6	9.1	11.3	19.0	30.2	35.6	44.0

Diabetes cases were defined as individuals reporting having been told by a physician or health care provider that they have diabetes or high blood sugar levels^50^. Hypertension cases were defined as individuals reporting that a physician said to them that they had hypertension and who also report using medications “to lower blood pressure” or control hypertension prescribed by a health care provider. Obesity was a BMI above or equal to 30 kg/m^2^^51^. Weight and height were directly measured in all countries, but in Argentina, it was self-reported. Tobacco was defined as current smoking in individuals who had at least smoked 100 cigarettes in their lifetime.

Using these definitions, the SALURBAL study computed prevalences by the city following an approach adapted from Quick et al.^52^. We estimated country-specific Bayesian hierarchical models to estimate sex-specific associations between each risk factor and 10-year age categories; models included sex-specific random intercepts for each city. Model estimates were used to obtain the prevalence of each risk factor for each age and sex group in each city. Age-standardized sex-specific prevalence estimates for each city were then obtained by weighting sex and age-specific estimates using the age distribution of all SALURBAL cities in 2010 to obtain city-specific estimates that are adjusted to the same age distribution and are therefore comparable across sexes, cities, and countries.

Data on population sizes by year and sex were obtained from intercensal population estimates or population projections from national census bureaus. We aligned the survey year with the year of city-level population sizes. For instance, we utilized the population size from 2013 for Argentina because it corresponded with the ENFR survey conducted in 2013. All data used in this study is public. Data is available at https://data.lacurbanhealth.org/.

### Statistical analysis

To calculate the number of prevalent cases in each city and risk factor, we multiplied the prevalence of each risk factor by the city’s population in that year. To estimate the scaling properties of each risk factor, we estimated a regression model of the form:

1 $$ln\left( {Y_{{ij}} } \right) = \alpha _{j} + \beta _{j} *ln\left( {N_{{ij}} } \right) + \epsilon _{{ij}}$$

Where $\:{Y}_{ij}$ is the number of cases of disease *j* (e.g., diabetes) in city *i*, and *Ni* is the population size of the city *i*, ɑ is a constant (intercept) and e is a residual. The *β* coefficients are the scaling coefficients, showing whether the different health outcomes are sub-linear (more likely to be found in smaller cities), linear (equally likely), or superlinear (more likely to be found in larger cities). Scaling coefficients (β) indicate whether the prevalence of these risk factors increases at a slower (sublinear, β < 1), equal (linear, β = 1), or faster (superlinear, β > 1) rate compared to the urban population size of cities. Confidence intervals (CI) provide a range of values within which the actual scaling coefficient is expected to fall.

To account for possible different levels of prevalence rates and cases by country and to further control for the role of age distribution in prevalence, we also expanded the model as follows:

2 $$\log ~\left( {Y_{{ij}} } \right) = \alpha + \beta ~ \cdot \log \left( {N_{{ij}} } \right)~ + \alpha _{2} \cdot Country_{i} + \alpha _{3} \cdot Prop\left( {15_{{39}} } \right)_{{ij}} + \alpha _{4} \cdot Prop\left( {40_{{64}} } \right)_{{ij}} + \alpha _{5} \cdot Prop\left( {65p} \right)_{{ij}} + \epsilon _{{ij}}$$

The vector of variables per country refers to the country where the city is located, and Prop(15_39), Prop(40_64), and Prop(65p) represent the percentage of the population in each city aged 15 to 39, 40 to 64, and 65+. We conducted these regressions in 2 different ways: (i) Unadjusted model: entire model with all cities and no country-level effects, allowing for the examination of the relation between city size and the outcome pooling across all countries; (ii) Adjusted model: with fixed effects for the country for the entire population combined and stratified by gender allowing for the examination of whether the patterns observed above persist after adjustment for country or vary by gender, both models age-adjusted. To determine the statistical significance of our findings, we calculated 95% confidence intervals for the scaling coefficients. Statistical analyses were conducted using the Python programming language, with the support of numpy, pandas, and sci-kit-learn libraries. All methods used in this study were performed under relevant guidelines and regulations, ensuring compliance with ethical and scientific standards.

## Conclusion

Our results add evidence to the distribution of CVD risk factors in many cities in Latin America. Overall, we found weak variations of the scaling coefficients around linearity, although sublinear scaling was observed for some risk factors, such as tobacco. Cultural attitudes toward CVD risk factors, urbanization, and population growth can vary significantly between countries. In larger cities, where urbanization often accelerates lifestyle changes, tobacco smoking may be more socially accepted or even encouraged, leading to higher prevalence rates. In contrast, in others, it may be stigmatized or heavily regulated. Further investigations of the scaling properties of risk factors and the causes of different scaling properties using longitudinal data may provide essential insights for policies to prevent CVD as cities grow.

## Electronic supplementary material

Below is the link to the electronic supplementary material.

Supplementary Material 1

## Data Availability

All data used in this study is public. Harmonized data will be made available on request. Please contact: salurbal@drexel.edu.
