# Supplementary material for: Scaling of cardiovascular risk factors in 230 Latin American cities
Source: Sci Rep. 2025 Mar 1;15:7279. doi: 10.1038/s41598-025-92087-5 (PMC11873263; doi:10.1038/s41598-025-92087-5)
Supplement: Supplementary file 1 — Supplementary Material 1 [file 41598_2025_92087_MOESM1_ESM.docx]

Figure SM1 – Map with the cities included in the study


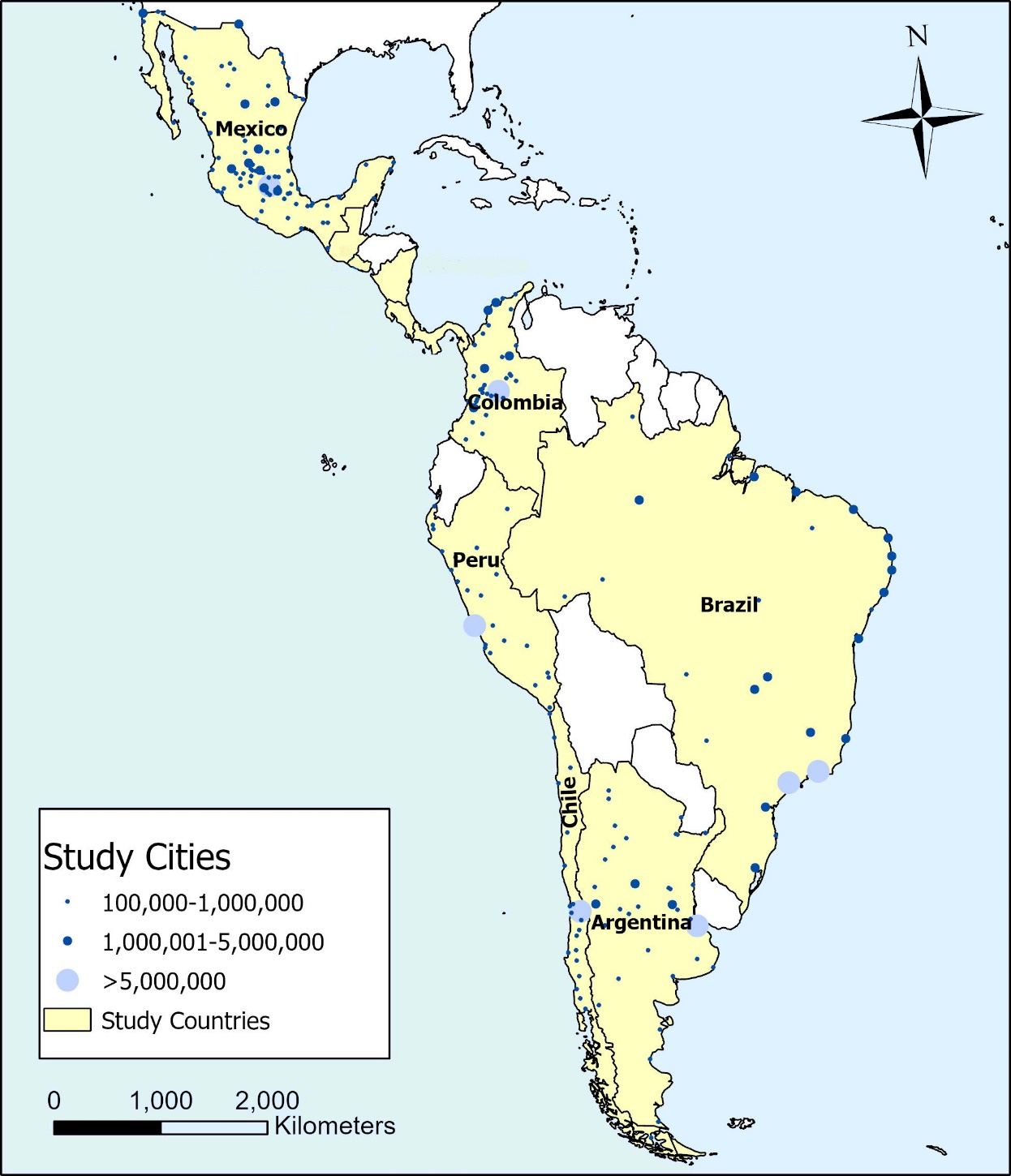


Source: Author’s own. * Map built using country boundaries from NaturalEarthdata.com and the city locations were created by the SALURBAL Built Environment Core. Creative Commons Attribution 4.0 International (CC BY 4.0).

Table SM1 – List of cities included in the study

| **Argentina, n=33** | | | | | | | |  | | |  | | | | | | |  |  |
| --- | --- | --- | --- | --- | --- | --- | --- | --- | --- | --- | --- | --- | --- | --- | --- | --- | --- | --- | --- |
| Bahia Blanca | | | Buenos Aires | Neuquen-Plottier-Cipolletti | | | | Rawson-Trelew | | |  |  |  |  |  |  |  |  |  |
| Mar del Plata | | | Concordia | San Carlos de Bariloche | | | | Rio Gallegos | | |  |  |  |  |  |  |  |  |  |
| San Nicolas de los Arroyos | | | Parana | Salta | | | | Zarate-Campana | | |  |  |  |  |  |  |  |  |  |
| Tandil | | | Formosa | Santiago del Estero | | | |  | | |  |  |  |  |  |  |  |  |  |
| Catamarca | | | Jujuy | Rosario | | | |  | | |  |  |  |  |  |  |  |  |  |
| Cordoba | | | Santa Rosa | Santa Fe | | | |  | | |  |  |  |  |  |  |  |  |  |
| Rio Cuarto | | | La Rioja | San Juan | | | |  | | |  |  |  |  |  |  |  |  |  |
| Resistencia | | | Mendoza | San Luis | | | |  | | |  |  |  |  |  |  |  |  |  |
| Comodoro Rivadavia | | | San Rafael | Villa Mercedes | | | |  | | |  |  |  |  |  |  |  |  |  |
| Corrientes | | | Posadas | San Miguel de Tucuman-Tafi Viejo | | | |  | | |  |  |  |  |  |  |  |  |  |
|  | | |  |  | | | |  | | |  |  |  |  |  |  |  |  |  |
| **Brazil, n=27** | | | | | | | |  | | |  | | | | | |  |  |  |
| Rio Branco | | | Cuiaba | Porto Alegre | | | |  | | |  |  |  |  |  |  |  |  |  |
| Maceio | | | Campo Grande | Porto Velho | | | |  | | |  |  |  |  |  |  |  |  |  |
| Macapa | | | Belo Horizonte | Boa Vista | | | |  | | |  |  |  |  |  |  |  |  |  |
| Manaus | | | Belem | Florianopolis | | | |  | | |  |  |  |  |  |  |  |  |  |
| Salvador | | | Joao Pessoa | Sao Paulo | | | |  | | |  |  |  |  |  |  |  |  |  |
| Fortaleza | | | Curitiba | Aracaju | | | |  | | |  |  |  |  |  |  |  |  |  |
| Brasilia | | | Recife | Palmas | | | |  | | |  |  |  |  |  |  |  |  |  |
| Vitoria | | | Teresina |  | | | |  | | |  |  |  |  |  |  |  |  |  |
| Goiania | | | Rio de Janeiro |  | | | |  | | |  |  |  |  |  |  |  |  |  |
| Sao Luis | | | Natal |  | | | |  | | |  |  |  |  |  |  |  |  |  |
|  | | |  |  | | | |  | | |  |  |  |  |  |  |  |  |  |
| **Chile, n=21** | | | | | | | |  | | |  | | | | |  |  |  |  |
| Arica | | | Rancagua |  | | | |  | | |  |  |  |  |  |  |  |  |  |
| Iquique | | | Talca |  | | | |  | | |  |  |  |  |  |  |  |  |  |
| Antofagasta | | | Curico |  | | | |  | | |  |  |  |  |  |  |  |  |  |
| Calama | | | Concepcion |  | | | |  | | |  |  |  |  |  |  |  |  |  |
| Copiapo | | | Chillan |  | | | |  | | |  |  |  |  |  |  |  |  |  |
| La Serena-Coquimbo | | | Los Angeles |  | | | |  | | |  |  |  |  |  |  |  |  |  |
| Valparaiso-Vina del Mar | | | Temuco |  | | | |  | | |  |  |  |  |  |  |  |  |  |
| Quillota | | | Valdivia |  | | | |  | | |  |  |  |  |  |  |  |  |  |
| San Antonio | | | Osorno |  | | | |  | | |  |  |  |  |  |  |  |  |  |
| Santiago | | | Puerto Montt |  | | | |  | | |  |  |  |  |  |  |  |  |  |
|  | | | Punta Arenas |  | | | |  | | |  |  |  |  |  |  |  |  |  |
|  | | |  |  | | | |  | | |  |  |  |  |  |  |  |  |  |
| **Colombia, n=35** | | | | | | | |  | | |  | | | |  |  |  |  |  |
| Apartado | | | Quibdo | Pereira | | | | Duitama | | |  |  |  |  |  |  |  |  |  |
| Medellin | | | Monteria | Barrancabermeja | | | | Girardot | | |  |  |  |  |  |  |  |  |  |
| Barranquilla | | | Bogota | Bucaramanga | | | | Fusagasuga | | |  |  |  |  |  |  |  |  |  |
| Cartagena | | | Neiva | Sincelejo | | | | Buga | | |  |  |  |  |  |  |  |  |  |
| Tunja | | | Riohacha | Ibague | | | | Sogamoso | | |  |  |  |  |  |  |  |  |  |
| Manizales | | | Santa Marta | Buenaventura | | | |  | | |  |  |  |  |  |  |  |  |  |
| Florencia | | | Villavicencio | Cali | | | |  | | |  |  |  |  |  |  |  |  |  |
| Yopal | | | Pasto | Cartago | | | |  | | |  |  |  |  |  |  |  |  |  |
| Popayan | | | Cucuta | Palmira | | | |  | | |  |  |  |  |  |  |  |  |  |
| Valledupar | | | Armenia | Tulua | | | |  | | |  |  |  |  |  |  |  |  |  |
|  | | |  |  | | | |  | | |  |  |  |  |  |  |  |  |  |
|  | | |  |  | | | |  | | |  |  |  |  |  |  |  |  |  |
| **Peru, n=23** | | | | | | | |  | | |  | | |  |  |  |  |  |  |
| Chimbote | | | Huancayo | Tacna | | | |  | | |  |  |  |  |  |  |  |  |  |
| Huaraz | | | Chiclayo | Tumbes | | | |  | | |  |  |  |  |  |  |  |  |  |
| Arequipa | | | Lima | Pucallpa | | | |  | | |  |  |  |  |  |  |  |  |  |
| Ayacucho | | | Trujillo |  | | | |  | | |  |  |  |  |  |  |  |  |  |
| Cajamarca | | | Iquitos |  | | | |  | | |  |  |  |  |  |  |  |  |  |
| Cusco | | | Piura |  | | | |  | | |  |  |  |  |  |  |  |  |  |
| Huanuco | | | Sullana |  | | | |  | | |  |  |  |  |  |  |  |  |  |
| Chincha Alta | | | Juliaca |  | | | |  | | |  |  |  |  |  |  |  |  |  |
| Ica | | | Puno |  | | | |  | | |  |  |  |  |  |  |  |  |  |
| Pisco | | | Tarapoto |  | | | |  | | |  |  |  |  |  |  |  |  |  |
|  | | |  |  | | | |  | | |  |  |  |  |  |  |  |  |  |
| **Mexico, n=91** | | | | | | | | | | |  | |  | | | | | | |
| Aguascalientes | Delicias | | | | Colima | Leon | | | | Mexico City | | Monterrey | | | | | | |  |
| Ensenada | Hidalgo del Parral | | | | Manzanillo | Uriangato | | | | Tianguistenco | | Oaxaca de Juarez | | | | | | |  |
| Mexicali | San Cristobal de las Casas | | | | Tecoman | Salamanca | | | | Toluca | | San Juan Bautista Tuxtepec | | | | | | |  |
| Tijuana | Tapachula | | | | Durango | San Francisco del Rincon | | | | La Piedad | | Santo Domingo Tehuantepec | | | | | | |  |
| La Paz | Tuxtla Gutierrez | | | | Acapulco de Juarez | Pachuca de Soto | | | | Morelia | | Puebla de Zaragoza | | | | | | |  |
| Campeche | Acuna | | | | Chilpancingo | Tula de Allende | | | | Uruapan | | Tehuacan | | | | | | |  |
| Ciudad del Carmen | Monclova | | | | Iguala | Tulancingo de Bravo | | | | Zamora | | Queretaro | | | | | | |  |
| Chihuahua | Piedras Negras | | | | Celaya | Guadalajara | | | | Cuautla | | San Juan del Rio | | | | | | |  |
| Juarez | Saltillo | | | | Guanajuato | Ocotlan | | | | Cuernavaca | | Cancun | | | | | | |  |
| Cuauhtemoc | Torreon | | | | Irapuato | Puerto Vallarta | | | | Tepic | | Chetumal | | | | | | |  |
| Playa del Carmen | | Navojoa | | | Acayucan | | Zacatecas | |  |  |  |  |  |  |  |  |  |  |  |
| Culiacan | | Nogales | | | Coatzacoalcos | |  | |  |  |  |  |  |  |  |  |  |  |  |
| Los Mochis | | San Luis Rio Colorado | | | Cordoba | |  | |  |  |  |  |  |  |  |  |  |  |  |
| Mazatlan | | Villahermosa | | | Minatitlan | |  | |  |  |  |  |  |  |  |  |  |  |  |
| Ciudad Valles | | Victoria | | | Orizaba | |  | |  |  |  |  |  |  |  |  |  |  |  |
| Rio Verde | | Matamoros | | | Poza Rica de Hidalgo | |  | |  |  |  |  |  |  |  |  |  |  |  |
| San Luis Potosi | | Nuevo Laredo | | | Veracruz | |  | |  |  |  |  |  |  |  |  |  |  |  |
| Obregon | | Reynosa | | | Xalapa | |  | |  |  |  |  |  |  |  |  |  |  |  |
| Guaymas | | Tampico | | | Merida | |  | |  |  |  |  |  |  |  |  |  |  |  |
| Hermosillo | | Tlaxcala | | | Fresnillo | |  | |  |  |  |  |  |  |  |  |  |  |  |

Figure SM2 – Scatterplots of cardiovascular risk factors and urban population size by sex for cities in six Latin American countries, unadjusted model

| *Entire population plots*  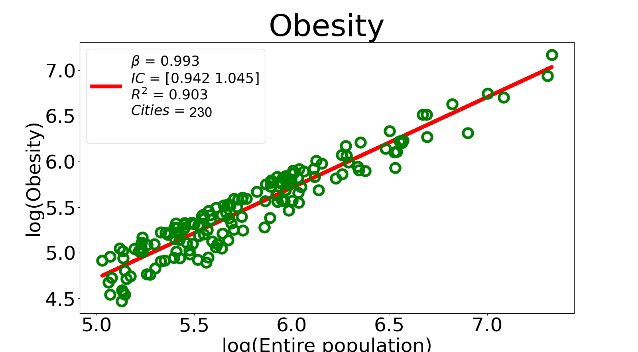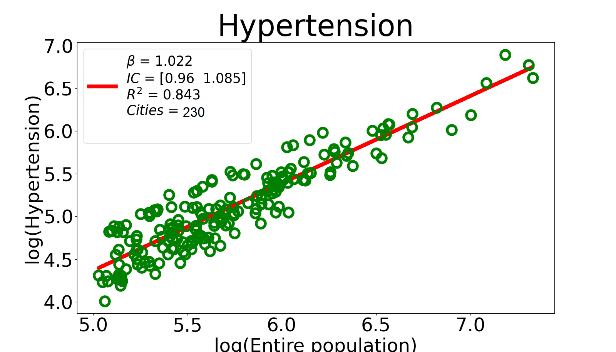  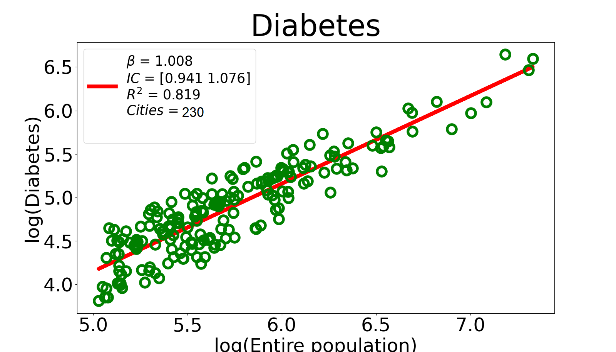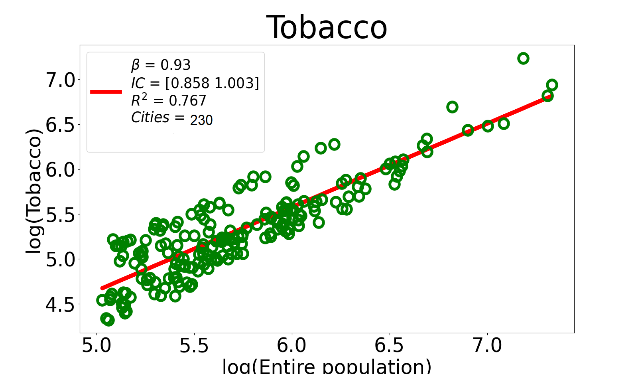 |
| --- |
| *Male population plots*  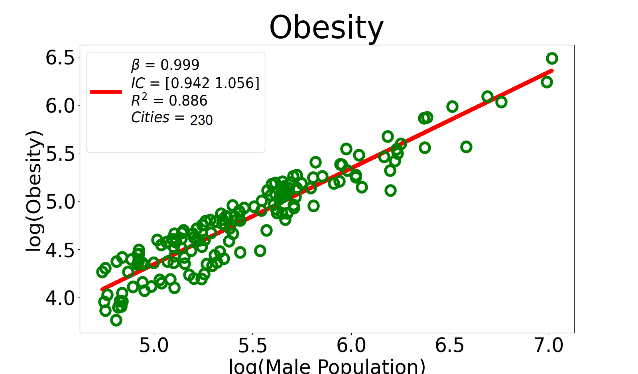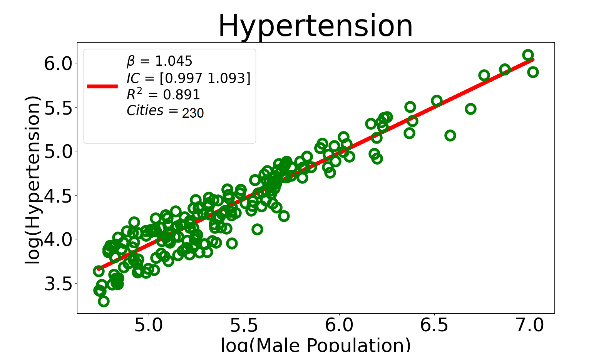  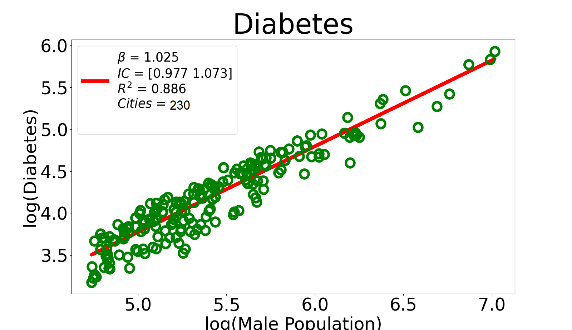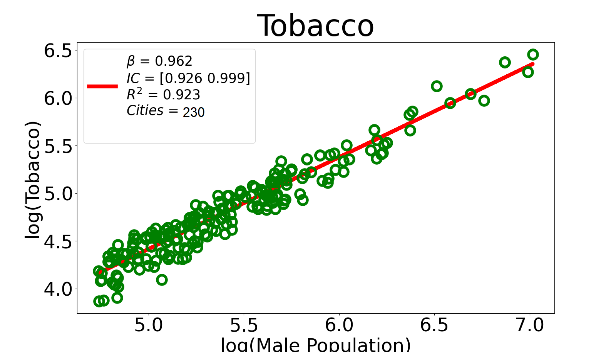 |
| 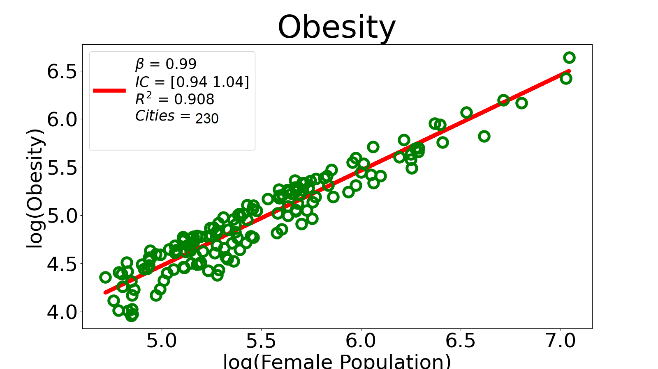*Female population plots*  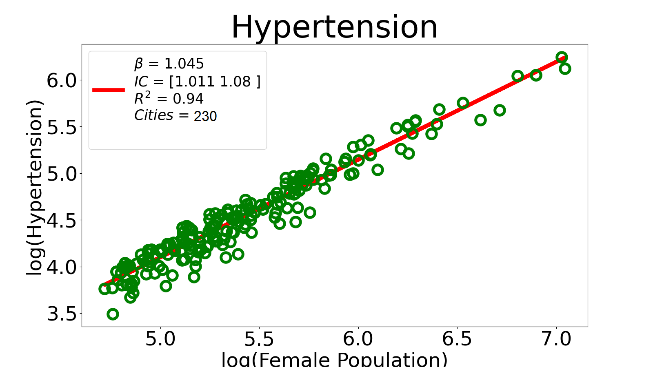  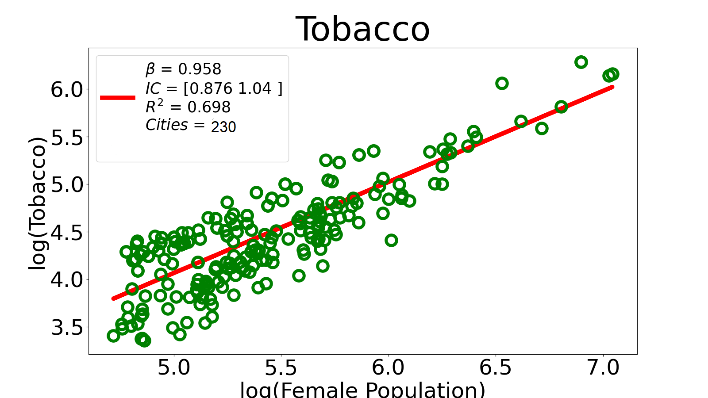  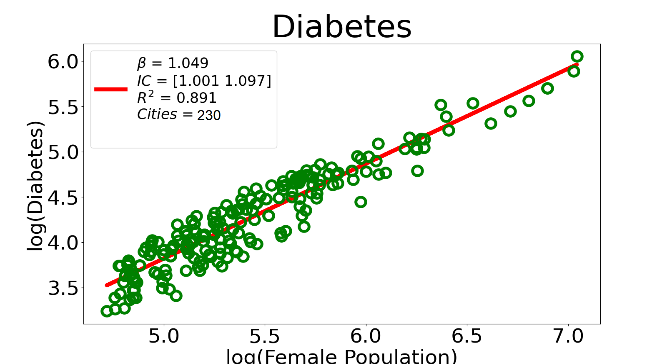 |

Source: Author’s own.

Figure SM3. Scaling coefficients values for risk factors by sex and country.

| 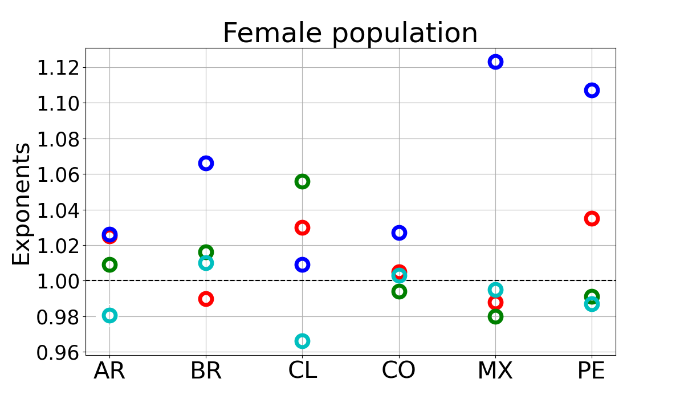 | 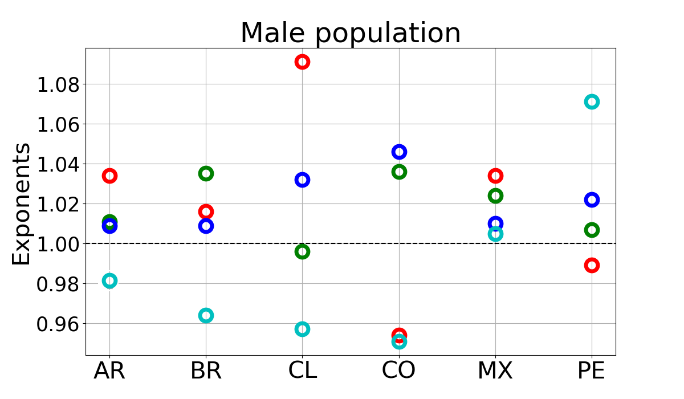 |
| --- | --- |
| 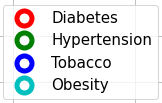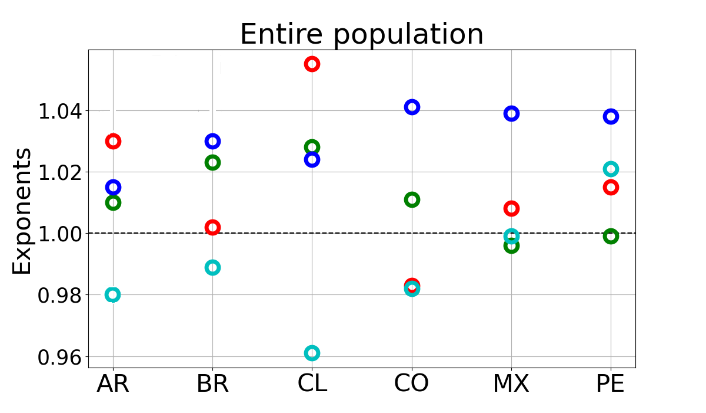 | |

*AR (Argentina), Brazil (BR), Chile (CL), Colombia (CO), Mexico (MX), Peru (PE).

Source: Author’s own.
